# Supplementary material for: Reverse evolution leads to genotypic incompatibility despite functional and active site convergence
Source: eLife. 2015 Aug 14;4:e06492. doi: 10.7554/eLife.06492 (PMC4579389; doi:10.7554/eLife.06492)
Supplement: Supplementary file 1. — Description of the directed evolution rounds. DOI: http://dx.doi.org/10.7554/eLife.06492.019 [file elife06492s005.docx]

*Supplementary file 1 for Kaltenbach at al., Reverse evolution leads to genotypic incompatibility despite functional and active-site convergence*

**Description of the directed evolution rounds**

** Supplementary File 1A.** Library creation and screening conditions for rounds 19-22 of the forward evolution and all rounds of the reverse evolution. Rounds 1-18 have been described in (Tokuriki et al., 2012).

[a] To avoid cross-contamination between libraries, the second trajectory library was placed into a pET-Strep vector with kanamycin resistance from round rev3b. Additionally, the *Hind*III restriction site was replaced by a *Kpn*I site from round rev5b to prevent cross-contamination during cloning.

[b] Fluoresceinyl-DETP: O-fluoresceinyl-O,O-diethyl-thiophosphate, Me-DEPCyC: 7-O-diethylphosphoryl-3-cyano-4-methyl-7-hydroxycoumarin.

**Information about Supplementary Files 1B-V.** These files give an overview of all selected clones. For activity measurements, cells were grown in triplicate. Cell lysates were sufficiently diluted to determine initial rates using paraoxon and/or 2NH as substrates, normalized to cell density (determined by absorption at 600 nm) and averaged. Activities are given relative to the activity of the parent mutant (columns “2NH” and “paraoxon”). The activity of the parent mutant is shown in the first row of each table in italics. Specificity for 2NH (column “2NH/paraoxon”) is given as the ratio of 2NH activity to paraoxon and is not normalized to the parent mutant. Chaperone dependence (column “+GroEL/-GroEL”) is the ratio of the activity measured in the presence of GroEL/ES to the activity in the absence of GroEL/ES and is also not normalized. Note that data from different tables is not directly comparable as lysates were diluted to different extents in different rounds (ranging from no dilution to 5 × 10^4^-fold) depending on the activity of the variants. Mutations are given relative to the parent mutant unless otherwise indicated. Enriched mutations are shown in grey boxes. For the reverse evolution, all changes in previously mutated positions are shown in bold, and back-to-ancestor mutations are additionally written in small, italic letters.

***Forward evolution***

**Round 1-18.** These rounds have been described in detail in the Supporting Information of reference (Tokuriki et al., 2013).

**Round 19.** The library was created by error-prone PCR from PTE-R18 using mutazyme and screened for increased specificity for 2NH.

**Supplementary File 1B.** Activity, selectivity (2NH/paraoxon), and mutations of round 19 variants.

 [a] This variant contained only synonymous mutations.

**Round 20.** The library was created by DNA shuffling of the 10 round 19 variants (**Supplementary File 1B**) and screened for increased specificity for 2NH.

**Supplementary File 1C.** Activity (relative to R19), selectivity (2NH/paraoxon), and mutations (relative to R18) of round 20 variants.

**Round 21.** The library was created by error-prone PCR from PTE-R20 using mutazyme and screened for increased specificity for 2NH.

**Supplementary File 1D.** Activity, selectivity (2NH/paraoxon), chaperone dependence (+GroEL/-GroEL), and mutations of round 21 variants.

 [a] n.d.: Activity in the absence of GroEL/ES was not detected; therefore, chaperone dependence could not be determined.

[b] This variant contained only synonymous mutations.

**Round 22.** The library was created by error-prone PCR from PTE-R21 using mutazyme and screened for reduced chaperone dependence.

** Supplementary File 1E.** Activity, selectivity (2NH/paraoxon), chaperone dependence (+GroEL/-GroEL), and mutations of round 22 variants.

[a] These variants contained only synonymous mutations.

**Round 23-25.** Three additional rounds of forward evolution were performed, but no variants with further reduced phosphotriesterase activity could be found without compromising arylesterase activity.

***Reverse evolution***

**Round rev1.** The library was created by error-prone PCR from AE using mutazyme and screened for increased phosphotriesterase activity.

** Supplementary File 1F.** Phosphotriesterase activity and mutations of round rev1 variants.

**Round rev2.** The library was created by DNA shuffling of 9 round rev1 variants (**Supplementary File 1F**) and 4 round 25 variants (**Supplementary File 1G**) and screened for increased phosphotriesterase activity.

**Supplementary File 1G.** Activity, selectivity (2NH/paraoxon), and mutations of round 25 variants.

**Supplementary File 1H.** Phosphotriesterase activity and mutations of round rev2 variants.

**Round rev3.** The library was created by error-prone PCR from PTE-revR2 using mutazyme and screened for increased phosphotriesterase activity.

**Supplementary File 1I.** Phosphotriesterase activity and mutations of round rev3 variants.

**Round rev4.** The library was created by DNA shuffling of 13 round rev3 variants (**Supplementary File 1I**) and screened for increased phosphotriesterase activity.

**Supplementary File 1J.** Phosphotriesterase activity (relative to revR3) and mutations (relative to revR2) of round rev4 variants.

**Round rev5.** The library was created by error-prone PCR from PTE-revR4 using mutazyme and screened for reduced chaperone dependence.

** Supplementary File 1K.** Phosphotriesterase activity, chaperone dependence (+GroEL/-GroEL), and mutations of round rev5 variants.

**Round rev6.** The library was created by DNA shuffling of the 7 round rev5 variants shown in **Supplementary File 1K** and an additional 10 unsequenced variants and screened for reduced chaperone dependence.

**Supplementary File 1L.** Phosphotriesterase activity (relative to revR5), chaperone dependence (+GroEL/-GroEL), and mutations (relative to revR4) of round rev6 variants.

**Round rev7.** The library was created by error-prone PCR from PTE-revR6 using mutazyme and screened for increased phosphotriesterase activity.

**Supplementary File 1M.** Phosphotriesterase activity and mutations of round rev7 variants.

[a] These variants contained only synonymous mutations.

**Round rev8.** The library was created by error-prone PCR from PTE-revR7a and PTE-revR7b using deoxynucleotide analogues and screened for increased phosphotriesterase activity.

** Supplementary File 1N.** Phosphotriesterase activity (relative to revR7a) and mutations (relative to revR6) of round rev8 variants.

**Round rev9.** The library was created by error-prone PCR from 5 revR8 variants (rev8_1, 3, 5, revR8a, and revR8b) using deoxynucleotide analogues and screened for increased phosphotriesterase activity.

** Supplementary File 1O.** Phosphotriesterase activity (relative to revR8b) and mutations (relative to revR6) of round rev9 variants.

**Round rev10.** The next library was made by error-prone PCR from PTE-revR9 using deoxynucleotide analogues. Because no improved variants were identified, a pool of the best 228 clones (with phosphotriesterase activities ≥ 0.5-fold relative to PTE-revR9) was taken into the next round.

**Round rev11.** Error-prone PCR (deoxynucleotide analogues) was performed on top of the plasmid mix from round rev10 and screened for reduced chaperone dependence. Again, no improved variants were identified, and 135 clones (with phosphotriesterase activities ≥ 0.5-fold relative to PTE-revR9) were taken into the next round.

**Round rev12.** Screening of a new error-prone library (deoxynucleotide analogues) using the plasmid mix from round rev11 yielded one variant with increased phosphotriesterase activity (1.3-fold relative to PTE-revR9), which contained the back-mutation V49A and a new mutation in a known position, I306M. This variant is “*neo*PTE”.

**Round rev13-14.** To corroborate that no further improvements could be found, a rev13 library was constructed by error-prone library (deoxynucleotide analogues) using a mix of 168 plasmids including *neo*PTE from round rev12. No significantly improved variants were found. The best variants had *neo*PTE-like activity and showed enrichment of I306M and a high fraction of synonymous mutations, indicating that no functional mutations could be found. To confirm whether the mutation V49*a* was also relevant or hitchhiking, *neo*PTE was back-shuffled with revR8x (round rev14). All variants kept the I306M mutation, and all but 1 the V49*a* reversion.

**Supplementary File 1P.** Phosphotriesterase activity and mutations of round rev13 variants.

 [a] This variant contained only synonymous mutations.

**Supplementary File 1Q.** Phosphotriesterase activity and mutations of round rev14 variants.

[a] Because mutations are given relative to *neo*PTE, the table shows that Ser258 and *Ala*49 were present in all but the indicated variants.

***Reverse evolution: Alternative trajectory excluding back-to-ancestor mutations***

In this trajectory, variants with improved phosphotriesterase activity were only accepted as templates for library generation for the next round if they did not contain any back-to-ancestor mutations.

**Round revR1b.** The first library was identical to round rev1. However, instead of shuffling selected clones, only PTE-revR1 was chosen for the next round as it exhibits the greatest increase in phosphotriesterase activity and lacks back-to-ancestor mutations.

**Round revR2b.** The library was created by error-prone PCR from PTE-revR1 using mutazyme and screened for increased phosphotriesterase activity.

**Supplementary File 1R.** Phosphotriesterase activity and mutations of round rev2b variants.

**Round revR3b.** The library was created by DNA shuffling of AE, rev2B_6 and revR2b, and screened for increased phosphotriesterase activity.

** Supplementary File 1S.** Phosphotriesterase activity (relative to revR2b) and mutations (relative to AE) of round rev3b variants.

**Round revR4b.** The library was created by error-prone PCR from revR3b, rev3b_2, and 7 using deoxynucleotide analogues and screened for reduced chaperone dependence.

**Supplementary File 1T.** Phosphotriesterase activity (relative to rev4b_0), chaperone dependence (+GroEL/-GroEL), and mutations (relative to AE+P135S+S308C) of round rev4b variants.

[a] Round rev4b was initially conducted in the presence of chaperones, but no improved variants were found. Because chaperone dependence was high in many clones, this round was repeated without chaperones and a clone from the first try with phosphotriesterase activity similar to revR3b and low chaperone dependence (“rev4b_0”) used for comparison.

**Round revR5b.** The library was created by error-prone PCR from PTE-revR4b using mutazyme and screened for increased phosphotriesterase activity.

** Supplementary File 1U.** Phosphotriesterase activity and mutations of round rev5b variants.

**Round rev6b.** PTE-rev5b was used as template for error-prone PCR to give library rev6b. All clones with improved phosphotriesterase carried back-to-ancestor mutations (**Supplementary File 1V**). Library creation was repeated but again, the screen yielded no improved variants devoid of back-mutations (not shown). Therefore, this trajectory reached an activity plateau in round rev5b.

** Supplementary File 1V.** Phosphotriesterase activity, chaperone dependence (+GroEL/-GroEL), and mutations of round rev6b variants.

***References***

Tokuriki N, Jackson CJ, Afriat-Jurnou L, Wyganowski KT, Tang R, Tawfik DS. 2012. Diminishing returns and tradeoffs constrain the laboratory optimization of an enzyme. *Nature Communications* **3**:1257.
