# Supplementary material for: Reverse evolution leads to genotypic incompatibility despite functional and active site convergence
Source: eLife. 2015 Aug 14;4:e06492. doi: 10.7554/eLife.06492 (PMC4579389; doi:10.7554/eLife.06492)
Supplement: Supplementary file 2. — Kinetic parameters of all variants. DOI: http://dx.doi.org/10.7554/eLife.06492.020 [file elife06492s006.docx]

*Supplementary file 2 for Kaltenbach at al., Reverse evolution leads to genotypic incompatibility despite functional and active-site convergence*

**Kinetic parameters of all variants**

**Information about Supplementary Files 2A-G.** These files give an overview of the kinetic parameters of all PTE variants. *Lysate measurements:* Cells were grown in at least duplicate and lysates sufficiently diluted (~1-10,000-fold) to determine initial rates of paraoxon and/or 2NH hydrolysis at a substrate concentration of 200 μM, normalized to cell density, corrected for the dilution factor, and averaged. This experiment was repeated twice and the combined average determined. *Purified enzyme measurements:* Variants were purified in at least duplicate in a 96-well format and sufficiently diluted (~ 0.1 nM - 10 μM) to determine initial rates of paraoxon and/or 2NH hydrolysis at a substrate concentration of 200 μM, corrected for the dilution factor, and averaged. Selected variants were purified on a larger scale to determine initial rates over a range of substrate concentrations and calculate Michaelis Menten parameters. For back-to-wild-type mutations, wild-type amino acid is shown in lower case italics.

**Supplementary File 2A.** Enzymatic activities of selected variants from the forward and reverse evolution in lysate and using purified enzyme.

[a] Note that the kinetic parameters from variant *wt*PTE to R18-1 were published in (Tokuriki et al., 2012). [b] Cells were grown in at least duplicate and lysates sufficiently diluted to determine initial rates of paraoxon and/or 2NH hydrolysis at a substrate concentration of 200 μM, normalized to cell density, corrected for the dilution factor, and averaged. The experiment was repeated twice and the combined average determined. [c] For better comparison, *w*tPTE was repuried and remeasured in parallel with the reverse evolution variants (*wt*PTE-2). [d] For better comparison, PTE-R18 was repurified and remeasured in parallel with the subsequent variants (R18-2).

**Supplementary File 2B.** Kinetic parameters of *wt*PTE, AE, and *neo*PTE variants (**Figure 4-7**) in lysate and using purified enzyme.

[a] For comparison, the effect of each mutation is given in the same direction, e.g. A45*t* means the effect of introducing *Thr* into each background (AE-A45*t*, *neo*PTE-A45*t*). When the amino acid in question (*Thr* in this case) is already present in a certain background, the effect is calculated as its reintroduction after removal (reversion of *wt*PTE-*t*45A to *wt*PTE).

[b] Cells were grown in at least duplicate and lysates sufficiently diluted (~1-10,000-fold) to determine initial rates of paraoxon hydrolysis at a substrate concentration of 200 μM, normalized to cell density, and corrected for the dilution factor. This experiment was repeated twice and the average change relative to the respective parent variant (*wt*PTE, AE, and *neo*PTE) and the standard deviation were determined. A t-test was performed to obtain p-values. Only mutants with an average >1.3-fold difference from the respective parent mutant AND a p-value <0.05 are considered significant. Non-significant values are underlined. Note that the only >1.3 fold change with non-significant p-value is AE+T341*i* (1.4-fold, p-value 0.15).

[c] Variants were purified in at least duplicate in a 96-well format and sufficiently diluted to determine initial rates of paraoxon hydrolysis at a substrate concentration of 200 μM, corrected for the dilution factor, averaged and the standard deviation determined.

[d] Phosphotriesterase activity was too low to be determined.

[e] No standard deviation is given because only a single measurement is shown.

**Supplementary File 2C.** Kinetic parameters of additional variants made to determine mutational effects over the forward evolution in lysate and using purified enzyme. The mutational effects are given in **Figure 5** and **6** and were calculated by comparing each variant to its parent indicated in the name (e.g. R8+I138M to R8) and as shown in **Supplementary File 2D**. The effect of all mutations not listed could be calculated directly by comparing variants from the forward evolution as shown in **Supplementary File 2D**.

[a] Cells were grown in at least duplicate and lysates sufficiently diluted (~1-10,000-fold) to determine initial rates of paraoxon hydrolysis at a substrate concentration of 200 μM, normalized to cell density, and corrected for the dilution factor. This experiment was repeated twice and the average change relative to the respective parent variant AE and the standard deviation were determined. P-values are shown in **Supplementary File 2D**.

[b] Variants were purified in at least duplicate in a 96-well format and sufficiently diluted to determine initial rates of paraoxon hydrolysis at a substrate concentration of 200 μM, corrected for the dilution factor, averaged and the standard deviation determined.

**Supplementary File 2D.** Mutational effects over the forward evolution in lysate and using purified enzyme.

[a] Cells were grown in at least duplicate and lysates sufficiently diluted (~1-10,000-fold) to determine initial rates of paraoxon hydrolysis at a substrate concentration of 200 μM, normalized to cell density, and corrected for the dilution factor. This experiment was repeated twice and the average change relative to the respective parent variant (shown in the column “Calculation”) and the standard deviation were determined. A t-test was performed to obtain p-values. Only mutants with an average >1.3-fold difference from the respective parent mutant AND a p-value <0.05 are considered significant. Non-significant values are underlined. Note that *l*140M and *t*199I have a >1.3 fold effect but non-significant p-values.

**Supplementary File 2E.** Kinetic parameters of additional variants made to determine mutational effects over the reverse evolution in lysate and using purified enzyme. The mutational effects are given in **Figure 5** and **7** and were calculated by comparing each variant to its parent indicated in the name (e.g. revR3+T172I to revR3) and as shown in **Supplementary File 2F**. The effect of all mutations not listed could be calculated directly by comparing variants from the reverse evolution as shown in **Supplementary File 2F**.

[a] Cells were grown in at least duplicate and lysates sufficiently diluted (~1-10,000-fold) to determine initial rates of paraoxon hydrolysis at a substrate concentration of 200 μM, normalized to cell density, and corrected for the dilution factor. This experiment was repeated twice and the average change relative to the respective parent variant AE and the standard deviation were determined. P-values are shown in **Supplementary File 2F**.

[b] Variants were purified in at least duplicate in a 96-well format and sufficiently diluted to determine initial rates of paraoxon hydrolysis at a substrate concentration of 200 μM, corrected for the dilution factor, averaged and the standard deviation determined.

**Supplementary File 2F.** Mutational effects over the forward evolution in lysate and using purified enzyme.

[a] Cells were grown in at least duplicate and lysates sufficiently diluted (~1-10,000-fold) to determine initial rates of paraoxon hydrolysis at a substrate concentration of 200 μM, normalized to cell density, and corrected for the dilution factor. This experiment was repeated twice and the average change relative to the respective parent variant (shown in the column “Calculation”) and the standard deviation were determined. A t-test was performed to obtain p-values. Only mutants with an average >1.3-fold difference from the respective parent mutant AND a p-value <0.05 are considered significant. Non-significant values are underlined. Note that V49*a* and *s*258N have a >1.3 fold effect but non-significant p-values.

**Supplementary File 2G.** Kinetic parameters of variants made for combinatorial mutational analysis (**Figure 7**) in lysate and using purified enzyme.

[a] Cells were grown in at least duplicate and lysates sufficiently diluted (~1-10,000-fold) to determine initial rates of paraoxon hydrolysis at a substrate concentration of 200 μM, normalized to cell density, and corrected for the dilution factor. This experiment was repeated twice and the average change relative to the parent variant AE and the standard deviation were determined. A t-test was performed to obtain p-values. Only mutants with an average >1.3-fold difference from the respective parent mutant AND a p-value <0.05 are considered significant. Non-significant values are underlined.

[b] Fold changes between the single and double mutants of each series shown in **Figure 7B** (*e.g.*, AE+*p*135S differs by 4.3-fold from the double mutant AE+*p*135S+I138*m*, AE+I138*m* differs by 2.6-fold). A t-test was performed to obtain p-values. Note that the two mutants AE+F271*l*+*s*308C and AE+M272*l*+*s*308C have non-significant p-values compared to the “double mutant” in this series, AE+F271*l*+M272*l*+*s*308C.

[c] Values for AE+F271*l* are compared to the double mutant AE+F271*l*+F313*l*. Values for AE+*s*308C are compared to the double mutant AE+I306*f*+*s*308C.

[d] AE+F271*l*+*s*308C and AE+M272*l*+*s*308C are “single mutants” of the parent AE+*s*308C and values are compared to the “double mutant” AE+F271*l*+M272*l*+*s*308C.

[e] Variants were purified to determine initial rates of paraoxon hydrolysis over a range of substrate concentrations and calculate Michaelis Menten parameters.

**Supplementary File 2H.** Absorption wavelength λ, extinction coefficient ε_λ_, and leaving group pKa values for substrates used for kinetic analysis. Values were taken from (Khersonsky and Tawfik, 2005).

[a] Extinction coefficients are given for a pathlength of 0.58 cm, which results from a 200 μL reaction volume in a 96-well plate.

[b] 2NH was not used for linear free energy relationship analysis.

***References***

Khersonsky O, Tawfik DS. 2005. Structure-reactivity studies of serum paraoxonase PON1 suggest that its native activity is lactonase. *Biochemistry* **44**:6371-6382.

Tokuriki N, Jackson CJ, Afriat-Jurnou L, Wyganowski KT, Tang R, Tawfik DS. 2012. Diminishing returns and tradeoffs constrain the laboratory optimization of an enzyme. *Nature Communications* **3**:1257.
