# Supplementary material for: Reverse evolution leads to genotypic incompatibility despite functional and active site convergence
Source: eLife. 2015 Aug 14;4:e06492. doi: 10.7554/eLife.06492 (PMC4579389; doi:10.7554/eLife.06492)
Supplement: Supplementary file 3. — Crystallographic information. DOI: http://dx.doi.org/10.7554/eLife.06492.021 [file elife06492s007.docx]

*Supplementary file 3 for Kaltenbach at al., Reverse evolution leads to genotypic incompatibility despite functional and active-site convergence*

**Crystallographic information**

**Supplementary File 3A.** Data collection and refinement statistics for structures reported in this work.

[a] Values in parenthesis are for the highest-resolution shell.

[b[ *R*_merge(I)_ = (Σ*_hkl_* Σ*_j_* |*I_hkl,j_ -* 〈*I_hkl_*〉|)/(Σ*_hkl_* Σ*_j_ I_hkl,j_*) where 〈*I_hkl_*〉 is the average intensity of *j* symmetry-related observations of reflections with Miller indices *hkl*.

[c] CC_1/2_ = percentage of correlation between intensities from random half-datasets.

[d] *R*_work_ = Σ*_hkl_*|F_(obs)_-F_(calc)_|/Σ*_hkl_*|F_(obs)_|; 5% of the data that were excluded from the refinement were used to calculate *R*_free_.
